# Validation of a Laser Ranged Scanner-Based Detection of Spatio-Temporal Gait Parameters Using the aTUG Chair

**DOI:** 10.3390/s21041343

**Published:** 2021-02-13

**Authors:** Sebastian Fudickar, Jörn Kiselev, Christian Stolle, Thomas Frenken, Elisabeth Steinhagen-Thiessen, Sandra Wegel, Andreas Hein

**Affiliations:** 1 Assistance Systems and Medical Device Technology, Carl von Ossietzky University Oldenburg, 26129 Oldenburg, Germany; christian.stolle1@uni-oldenburg.de (C.S.); andreas.hein@offis.de (A.H.); 2 Geriatrics Research Group, Charité–Universitätsmedizin Berlin, Corporate Member of Freie Universität Berlin, Humboldt-Universität zu Berlin, and Berlin Institute of Health, Charitéplatz 1, D-10117 Berlin, Germany; joern.kiselev@charite.de (J.K.); elisabeth.steinhagen-thiessen@charite.de (E.S.-T.); sandra.wegel@charite.de (S.W.); 3 Department of Anesthesiology and Operative Intensive Care Medicine (CCM, CVK), Charité–Universitätsmedizin Berlin, Corporate Member of Freie Universität Berlin, Humboldt-Universität zu Berlin, and Berlin Institute of Health, Charitéplatz 1, D-10117 Berlin, Germany; 4 IT Services Thomas Frenken, Loyerweg 62a, 26180 Rastede, Germany; itservices@thomasfrenken.de; 5 Divison of Lipid Metabolism of the Department of Endocrinology and Metabolic Medicine, Charité–Universitätsmedizin Berlin, Corporate Member of Freie Universität Berlin, Humboldt-Universität zu Berlin, and Berlin Institute of Health, Charitéplatz 1, D-10117 Berlin, Germany; 6 Department of Surgery (CCM, CVK), Charité–Universitätsmedizin Berlin, Corporate Member of Freie Universität Berlin, Humboldt-Universität zu Berlin, and Berlin Institute of Health, Charitéplatz 1, D-10117 Berlin, Germany

**Keywords:** timed “Up & Go” test, TUG, laser ranged scanner, automated assessment, spatio-temporal gait parameter

## Abstract

This article covers the suitability to measure gait-parameters via a Laser Range Scanner (LRS) that was placed below a chair during the walking phase of the Timed Up&Go Test in a cohort of 92 older adults (mean age 73.5). The results of our study demonstrated a high concordance of gait measurements using a LRS in comparison to the reference GAITRite walkway. Most of aTUG’s gait parameters demonstrate a strong correlation coefficient with the GAITRite, indicating high measurement accuracy for the spatial gait parameters. Measurements of velocity had a correlation coefficient of 99%, which can be interpreted as an excellent measurement accuracy. Cadence showed a slightly lower correlation coefficient of 96%, which is still an exceptionally good result, while step length demonstrated a correlation coefficient of 98% per leg and stride length with an accuracy of 99% per leg. In addition to confirming the technical validation of the aTUG regarding its ability to measure gait parameters, we compared results from the GAITRite and the aTUG for several parameters (cadence, velocity, and step length) with results from the Berg Balance Scale (BBS) and the Activities-Specific Balance Confidence-(ABC)-Scale assessments. With confidence coefficients for BBS and velocity, cadence and step length ranging from 0.595 to 0.798 and for ABC ranging from 0.395 to 0.541, both scales demonstrated only a medium-sized correlation. Thus, we found an association of better walking ability (represented by the measured gait parameters) with better balance (BBC) and balance confidence (ABC) overall scores via linear regression. This results from the fact that the BBS incorporates both static and dynamic balance measures and thus, only partly reflects functional requirements for walking. For the ABC score, this effect was even more pronounced. As this is to our best knowledge the first evaluation of the association between gait parameters and these balance scores, we will further investigate this phenomenon and aim to integrate further measures into the aTUG to achieve an increased sensitivity for balance ability.

## 1. Introduction

The process of aging is accompanied by a loss of physical capabilities and, consequently, a loss of function [1]. While this process is, to a certain degree, inevitable, it is amplified by the often simultaneous presence of chronic diseases, a phenomenon termed “multimorbidity” [2]. Multimorbidity is related to frailty [3]. Frailty was first described by Fried et al. (2001) as a clinical syndrome with the presence of one or more of five defined conditions (unintentional weight loss, self-reported exhaustion, muscle weakness, slow walking speed and low physical activity) [4]. While the presence of one or two of these conditions is labelled as pre-frailty, the simultaneous presence of three or more conditions is defined as frailty. With a prevalence of up to 58%, frailty is a challenge in ageing societies [5].

Frailty is associated with several negative developments for older people, among them restrictions in activities of daily living (ADL) [6,7,8], cognitive decline [9,10], and lower levels of quality of life (QoL) [8,11]. Furthermore, frailty leads to a higher fall risk [12] and higher levels of fear of falling [13], both of whom are known to lead to a further decline in physical activity [14]. Additionally, frailty is associated with an increased risk of post-surgical complications [15,16], and overall mortality [17]. However, current evidence suggests that increasing physical activity (PA) can reverse frailty [18], a decline in physiological performance [18], and contributes to the prevention of falls [19,20].

Consequently, a sizable number of frailty assessments were developed [21], of which virtually all incorporate measurements of function and mobility due to the central role these factors play in the development and treatment of frailty. Therefore, measuring function and mobility plays a central role in detecting frailty in older people. Fried et al. used the term “slowness” in their approach to evaluate gait speed (GS) [4]. GS is an important functional parameter with lower speed being associated with disability, cognitive decline, falls, hospitalization and mortality [22,23]. However, GS as a single measurement factor for the identification of frailty lacks validity [24]. Consequently, more detailed analyses of specific gait parameters have been evaluated for their ability to detect functional decline and frailty. In this, factors such as cadence, as well as step and stride length have been discussed as potential parameters to identify older people with frailty or at risk of falls [25,26].

Another valid option to measure mobility in older people is the Timed Up&Go (TUG) test [27]. The TUG is a widespread instrument for mobility assessment in geriatrics and has been recommended for the management of frailty by the British Geriatrics Society [28] as well as for preoperative assessments of geriatric assessments [29]. When measuring TUG, duration to complete the TUG with longer times tend to indicate mobility restrictions [27].

Results from the TUG can predict functional decline and frailty in older people [30] and can be used as part of a frailty screening tool for predicting postoperative complications [31]. Additionally, subtasks of the TUG, such as chair-rising, sitting, walking, and turning, can be measured separately while performing the TUG to enhance measurement precision as proposed by Botolfsen et al. (2008) [32].

However, although the TUG is quick and easy to perform, it still requires a trained assessor. Automated measurements of the TUG, in comparison, would allow for repeated measurements e.g., in the living environment of an older person to be able to detect early signs of decline.

With the validity of the TUG for age-related functional-decline and fall risk being confirmed, various technical-supported test systems have been proposed and validated to measure TUG times and associated gait parameters. Technical support systems for the TUG can be categorized according to the type of sensors used [33]. Inertial sensors, placed at the feet or the hip, have been used to measure the TUG and their suitability is confirmed [34,35,36]. Also, depth image cameras (such as the Kinect) have been validated to measure gait-parameters such as number of steps (step-count), step length, step duration, cadence, and gait velocity to cluster fallers from non-fallers [37].

Generally, the use of technical support systems for the TUG demonstrate distinctive advantages beyond automated report generation. Dibble et al. (2006) reported an improved inter-tester reliability [38] when using automated measurements. Furthermore, as gait parameters such as cadence [39] or step length [40] can provide additional information on functional ability, automated systems should be able to identify such parameters. However, the use of such technical measurement systems have their own challenges. Camera-based systems have certain requirements to provide reliable data, such as a clear view and a minimum distance to the measurement subject [41]. Provision of such requirements can be quite challenging [42]. In contrast, the use of wearable devices provide their own challenges regarding handling [33]. Thus, technology-based systems for TUG measurements that avoid such challenges or provide easy-to-use solutions for these problems are still missing.

**Figure 1:**
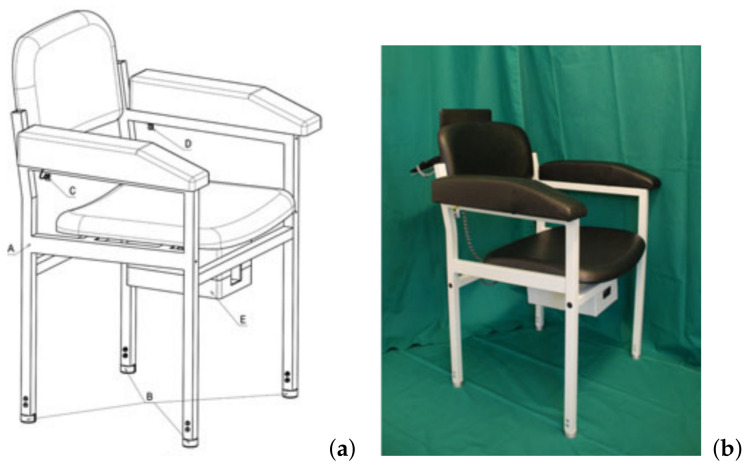
The aTUG chair as (**a**) conceptual design and (**b**) the prototype, used in the study.

Consequently, we have proposed an ambient measurement system to measure the TUG-the “ambient TUG (aTUG) chair” (see Figure 1a) [43]. The aTUG chair integrates light barriers (LB), force sensors (FS) and a Laser Range Scanner (LRS) into a standard chair used routinely in medical settings. Using these sensors, the chair can automatically measure the total duration of the TUG.

The aTUG’s validity to measure step length and step duration was initially confirmed within a trial with 7 participants [44]. In addition, the aTUG’s validity to measure total TUG duration via light barriers with a root mean squared error (RMSE) of 0.83 s and force sensors of 0.90 s was confirmed [45] in a clinical trial with 100 older adults with a mean age of 74 years. However, the validity to detect gait parameters via the integrated LRS for this geriatric cohort has not been confirmed yet.

For a similar LRS-based TUG system [46] the validity to measure walking speed (m/s), cadence (step/s), stride length (m), step length (m) and step width (m) has been evaluated with 7 young adults. Its validity to measure stride length and walking speed was confirmed in a pilot study with 16 older adults (mean age of 78.1 years) [47]. The system has been further-on evaluated regarding its suitability to detect mild cognitive impairment via a Mini-Mental State Examination (MMSE), using a cut-off value of 26, with 63 older adults (mean age 73.0 years) [48].

In addition, the validity to extract spatio-temporal gait parameters via LRS on a longer distance (e.g., a 4.8 m walking test) by applying LRS sensors at both ends of a walkway have been successfully evaluated in a study with ten healthy adults [49]. Therefore, as well the validity of the approach to extract stance, swing, stride, and double support times as well as stride and step-lengths were shown.

However, an evaluation of aTUG’s LRS sensor validity to measure spatio-temporal gait parameters and their correlation with well-established geriatric tests for a representative study cohort is still missing. To address this gap, this paper aims to validate the measured gait parameters in a representative geriatric cohort. Furthermore, we investigate the correlation among the resulting gait-parameters and well-established geriatric tests.

## 2. Materials and Methods

### 2.1. Composition of the aTUG Chair

The aTUG chair consists of a commercially available medical chair (Figure 1b), with integrated infrared light barriers (LBs) (as C and D), force sensors (FSs) (as B) (one at each leg of the chair) and a laser ranged scanner (LRS) (as part of E). The LRS is placed below the seating of the chair (Figure 1a). As LRS, a Hokuyo UTM-30LX is used which has a detection range of 0.1 to 30 m and 270 and scans in 0.25 steps with a frequency of 40 Hz per scan. The LRS measures distances to obstacles gradiently on the horizontal plane which is well suited to estimate leg positions. A control box (E) was located under the seat containing the amplifiers for the pressure sensors, a micro-controller board for signal processing and controlling the LB, a power supply and the LRS. Figure 1b shows the applied prototype. For the study, a visible marker was attached to the floor three metres from the chair.

### 2.2. Computation of aTUG Gait Parameters

**Figure 2:**
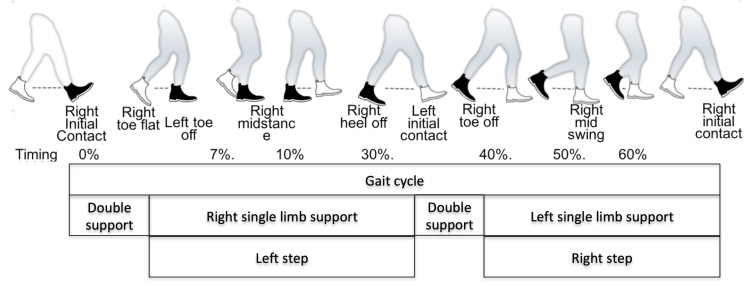
Gait cycle with corresponding events (along with a typical ratio-timing based on occurrence within a gait cycle) and corresponding phases; black shoe represents right foot (own depiction based on [52]).

The computation of the spatio-temporal gait parameters was conducted based on the work of Perry et al. (2010) [50] in accordance with the equations described by Frenken et al. (2012) [51]. The respective parameters are are summarized in Figure 2 and Table 1. With steps and stance/swing phases being calculated for each foot, additional parameters such as gait speed, cadence and step length were calculated accordingly.

The measured spatio-temporal gait parameters were then summarized by the aTUG system within a test-specific pdf (Latex) document, from where they were extracted for evaluation purposes.

### 2.3. Study Design and Recruitment

This study was designed as a cohort study with two measurement points at day one and day 30. A sample size calculation (SSC) was performed based on the method presented by Li and Fine [53] for comparing the sensitivity and specificity of the aTUG in respect of its ability to predict falls, with α = 0.05 and β = 0.2. Using this calculation, we needed 90 subjects and assumed a 10–20% dropout rate. We therefore aimed to recruit 110 participants.

Participants consisted of older adults aging 55+ years with mobility limitations (defined by a TUG of >15 s). Geriatric research often has a much higher minimum age defined; however, our aim was to achieve higher levels of variability in the measurements. For this reason, we defined 55 years as minimum age. Moreover, we included participants with mobility restrictions as they represent the target cohort during the development of the aTUG chair. In accordance with the available multifarious literature on TUG thresholds to detect mobility limitations, we selected a threshold of >15 s for our study as this was the threshold used in the geriatric hospital where the majority of participants were recruited. Recruitment took place in a large geriatric hospital, senior residences in the vicinity of the hospital, and by bulletins and emails sent to assisted-living facilities, providing details of the study. In the latter case, contact to the study team was initiated by the potential participants.

Verbal and written information was provided to all potential participants and all participants signed a written informed consent form before study measurements started. Inclusion and exclusion criteria were checked at the start of the first visit. In addition to age and mobility limitation, inclusion criteria for the study consisted of the ability to communicate verbally and to follow instructions, the ability to walk independently for six metres and to stand up (walking aids were allowed). Exclusion criteria were defined as the inability to walk or total dependence on a walking frame or walker, a body weight of more than 120 kg, severe affective or cognitive deficits according to medical records (Mini-Mental State Examination [MMSE] <24 and/or documented psychological conditions resulting in professional treatment or according to the responsible neuropsychologist), severe or unstable medical conditions, neurologic conditions and persons held in custody.

The trial received ethical approval from the ethics committee of Berlin and was registered at the Federal Institute for Drugs and Medical Devices (BfArM) and the German Institute of Medical Documentation and Information (DIMDI). The trial has the EUDAMED-number CIV-11-08-001887 and the DIMDI-number 00018377.

### 2.4. Measurements and Assessments

We planned 2 visits with all participants, with both visits being approximately 30 days apart from each other. At both visits the exact same measurement protocol was performed, consisting of the TUG and aTUG; and a gait analysis using the GAITRite^®^ walkway system were performed. Additional measurements were taken to evaluate balance ability as well as balance confidence, using the Berg Balance Scale (BBS), the Activities-Specific Balance Confidence-(ABC)-Scale, and the Tinetti Test (TT).

To investigate the validity of aTUG’s LBs and FSs to measure the overall TUG duration, TUG and aTUG tests were performed. TUG and aTUG-times were measured in parallel within one test performance of a participant, with each participant performing two laps. Participants started seated in a chair and stood up upon a signal. They then walked 3 m, turned around, returned to the chair, and sat down again. During both laps, the TUG was measured by an assessor using a stopwatch while the aTUG-times were recorded via the aTUG sensors simultaneously. Participants were instructed to walk at their comfortable walking pace while performing the TUG and they had one training lap before the actual measurement laps began. Between both measurement laps, participants had a rest of at least three minutes. The rest-duration was extended if the patient deemed it necessary or when showing visible signs of exertion. The findings of this study-aspect have been already reported in [45].

To study the validity of the spatio-temporal parameters as recorded by aTUG’s LRS, all participants completed two additional laps of synchronized measurements of a gait analysis using the GAITRite^®^ walkway system (CIR Systems Inc., Peekskill, NY, USA) and the aTUG chair. For this, the aTUG-chair was placed directly behind the GAITRite^®^. The GAITRite^®^ consists of a sensor-based floor mat able to measure gait parameters such as gait velocity and cadence, step time and length and single support /double support time ratio. The GAITRite^®^ is a widespread and valid tool for measuring gait parameters [54] with an excellent agreement of measurements in comparison with optical motion analysis systems such as Vicon, which must be considered to be the current gold standard for motion analysis [55]. During the gait analysis, participants started the measurement in front of the aTUG chair. Upon a signal, they started to walk along the walkway in their preferred gait speed until they left the walkway. The measurement was repeated after a short break. Both system times were synchronized in post-processing via a custom synchronisation-tool to provide synchronized timestamps. In contrast to the TUG-measurements, participants did not turn around but rather extended the walking phase to the end of the GAITRite^®^. This adjustment was necessary as the GAITRite^®^ was only able to measure unidirectional walks and the turning and walk-back phases could not be recorded by the GAITRite^®^. The measurement was repeated after a short break.

After completing the gait analysis, participants performed additional geriatric assessments:

- The Berg Balance Scale (BBS) is a 14-item test battery for assessing balance and mobility in older people. Each item is rated between 0 and 4 points, leading to total of 0 to 56 points [56].
- The Tinetti Test (TT) [57] measures static and dynamic balance ability and consists of 20 items that are either rated dichotomous or on a scale between 0 and 2 points. The total score of the TT has a range between 0 and 28 points.
- The Activities-Specific Balance Confidence-(ABC)-Scale [58] consists of 16 items asking participants about their confidence in performing certain activities of daily living without losing their balance. While the original scale asked participants to rate their confidence for each item as a percentage value, Filatrault et al. (2007) established a simplified version of the ABC-scale with a 0–3-point-Likert scale, with 3 points representing a participant being “very confident” to maintain balance in a given task and 0 points being “not at all confident” [59]. In this study, we used the validated German version of the ABC scale [60] with the 4-point-Likert scale. For all additional tests, higher results represent better functional ability and less disability.

Sociodemographic variables and present chronic conditions were documented. All assessments were performed according to a standardized protocol.

### 2.5. Data Pre-Processing and Statistical Analysis

Measurements of the aTUG SLR and the GAITRite^®^ were exported as csv files (covering measured velocity, cadence, and leg-specific averaged step lengths, stride lengths, stance times, and swing times per walk). To assure comparability among aTUG and GAITRite^®^ measures, for the aTUG only the steps of the initial walking-direction were considered. Per subject, a list of all walks measured by GAITRite^®^ and the aTUG was extracted from the respective export files. Recorded walks among both systems were matched based on the subject identifier and the synchronized times. Therefore, the clock-synchronisation was applied. Recordings of the walks among the GaitRite^®^ and the aTUG were treated as correctly synchronized if start times of recordings among both systems fell within an additional 2-min variation.

Per correctly synchronized walks, the gait parameters were calculated via aTUG’s signal-processing chain. Afterwards, the parameters’ error statistics were calculated as mean, SD, min, max and quartiles per considered walk. Within, the error was calculated via following formula was used: error = abs (GAITRite_value–aTUG_value)

Subsequently, each parameter was evaluated using the Spearman correlation coefficient of both systems as some variables were not normal distributed. In addition, Spearman correlation coefficients and scatter plots were calculated for the measured gait parameters and ABC and BBS scores, respectively.

The computations were conducted via Python and the NumPy and Pandas libraries.

## 3. Results

### 3.1. Study Sample

The included cohort consisted of 94 participants who recorded a total of 188 walks. Most of the participants conducted 2 walks as per protocol. However, the number of recorded walks per participant ranged between 1 and up to 5 walks. Out of the 188 walks, 24 recordings (12.7%) were compromised and could not be analysed. This excluded 5 participants (of the original included 99 participants) (5%) with no valid recordings from the study. Another 2 recordings were excluded because less than 4 steps could be measured, which is not enough for an acceptable analysis. For one of the examined participants, no valid ABC Score could be obtained. Therefore, this participant was not included in the correlation analysis of ABC and the measured gait parameters.

A total of 91 participants had at least one valid and matched walk recorded by both the GAITRite^®^ and aTUG. this cohort consisted of 64 females and 27 males with a mean age of 73.5 years. Table 2 describes the distribution of age, height, weight, BMI and the corresponding ABC and BBS scores among these participants.

### 3.2. Statistical Evaluation

#### 3.2.1. Sensitivity of Spatio-Temporal Gait Parameters of the aTUG chair

**Figure 3:**
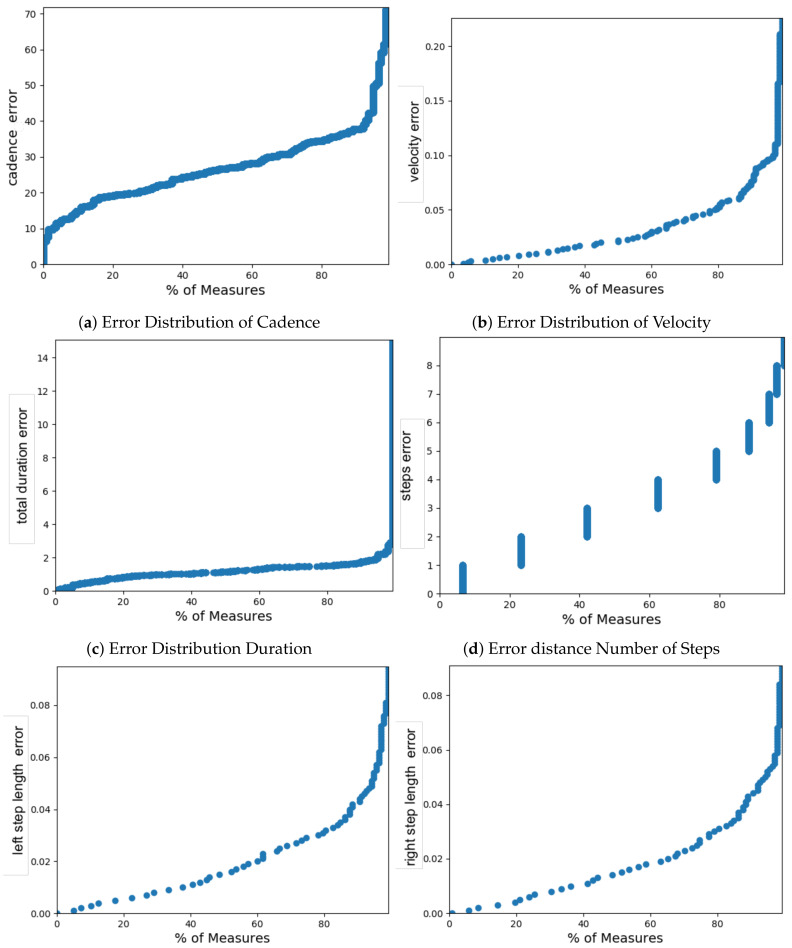
Error Distributions of Spatio-Temporal Parameters.

Gait measurements of all participants which were recorded by both the aTUG and GAITRite^®^ system were considered in the sensitivity analysis of the aTUG. The analysis included the following parameters: number of steps (count), total distance (m), total duration (s), Velocity (m/s), Cadence (steps/min), step length L (m), and step length R (m), stride length L (m), stride length R (m), stance time L (m), stance time R (m), swing time L (s), swing time R (s). Table 3 presents all parameters ordered by the correlation coefficient (CC). Parameters with a CC > 0.8 included the number of steps, total test duration, velocity, cadence, and step and stride length for the left and the right foot. In contrast, total distance as well as stance and swing time measurements revealed much lower results. Corresponding error distribution curves of relevant spatio-temporal parameters are shown in Figure 3.

#### 3.2.2. Influence of BBS and ABC Score on Spatio-Temporal Gait Parameters

**Figure 4:**
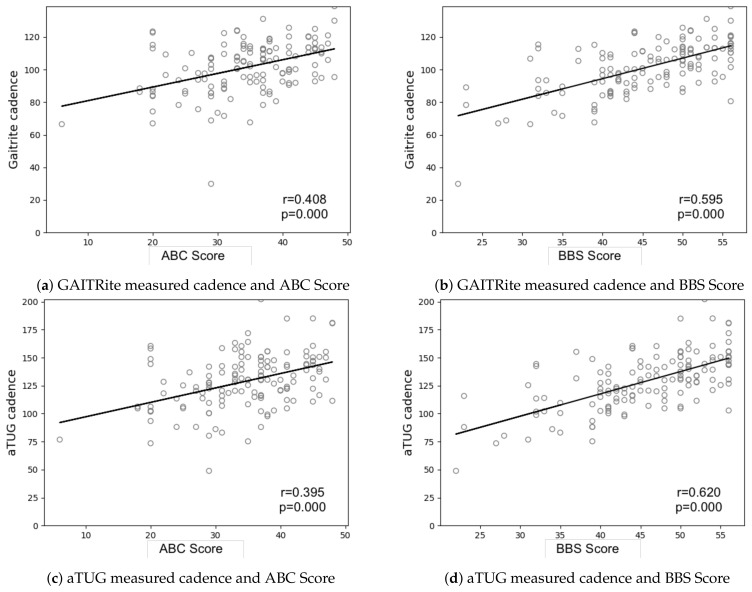
Scatterplot distribution among parameters.

**Figure 5:**
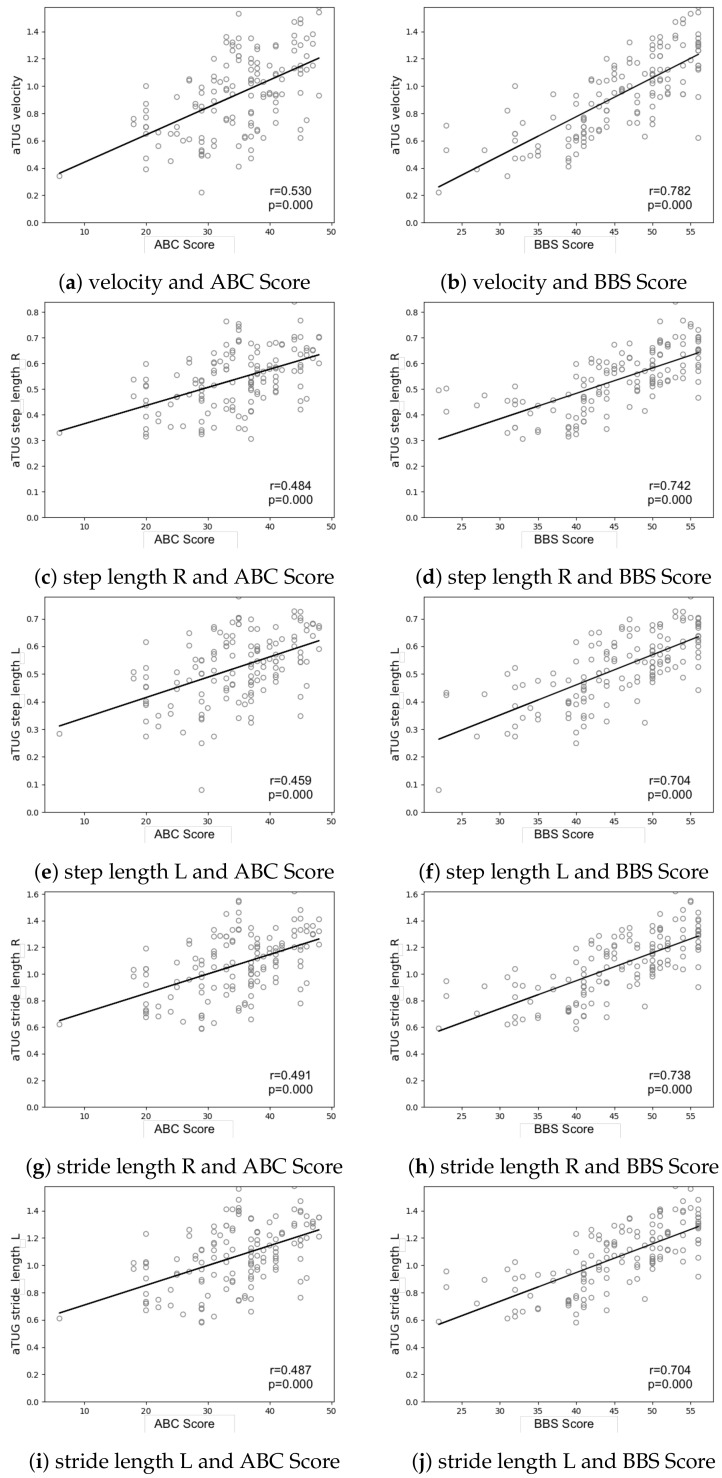
Scatterplot distribution among aTUG measured gait parameters, with ABC and BBS scores (partially leg-specific where applicable L = left, R = right leg).

The Berg Balance Scale (BBS) and Activities-Specific Balance Confidence Scale (ABC) were calculated for each participant. Their influence on the measured gait parameters for the GAITRite^®^ and aTUG measurements are depicted in Figure 4 and Figure 5. Both scores showed a similar trend, with the main difference being the offset of the y-axis. As could be expected, higher scores in the BBC and ABC were associated with better outcomes in the measured gait parameters. This trend was more pronounced for the BBS with R-Values between −0.787 and −0.611 for values that presented a negative correlation (step count, duration), and R-values from 0.595 to 0.798 for positive correlations (velocity, cadence, step, and stride length). R-values for the ABC-Scale measured between −0.506 and −0.566, and 0.395 and 0.541, respectively (Table 4).

## 4. Discussion

The results of our study demonstrated a high concordance of gait measurements using a laser range sensor attached to a chair in comparison to the reference GAITRite^®^ walkway. As shown in Table 3, most of aTUG’s gait parameters demonstrate a strong CC with the GAITRite^®^ system, indicating high measurement accuracy for the spatial gait parameters. This was additionally confirmed by the corresponding error-distribution curves shown in Figure 3. Measurements of velocity had a CC of 99%, which can be interpreted as an excellent measurement accuracy. Cadence showed a slightly lower CC of 96%, which is still a particularly good result, while step length demonstrated a CC of 98% for both legs individually. Interestingly, CC values for stride length was slightly better with an accuracy of 99% for each leg. This might be an indicator that stride length is a more robust measurement construct than step length, as it averages out potential variances during gait. Stride variability has been identified as an indicator for gait stability in a study using the GAITRite^®^ walkway system [61]. As the aTUG was able to measure stride length with similar precision, further steps in the development of the system should include the analysis of stride variability to account for this important parameter.

Considering the error distribution curves, overall a late error-increase could be observed with the notable exception of step-length and cadence, which demonstrated a more continuous increase of the measurement error. While it is entirely possible that this distribution could be observed due to a lack of measurement precision of the aTUG, another explanation is the measurement technology used in the reference system GAITRite^®^. As the GAITRite^®^ uses physical capacitive sensors, this results a measurement error of 1.27 centimetres (according to the technical reference sheet provided by the manufacturer) due to the corresponding size of the integrated capacitive pressure sensors. Therefore, the observed error distribution for step length might be distributed among both the reference system and the aTUG. As this inherent error of the reference system perfectly fits to the mean error (as shown in Table 3), a minimal error for the aTUG in measuring the step-length could be assumed. However, to confirm this hypothesis, other reference standards such as the camera-based Vicon system should be used.

Measurements of gait parameters are important components in the detection of frailty. In a systematic review by Buta et al. [21], they identified 67 instruments for the detection of frailty of which a majority used physical function and/or mobility as part of their evaluation. Fried et al. (2001), in the first publication describing the phenomenon of frailty [4], used walking time over 15 ft. for evaluating mobility. Walking speed as well as the TUG can be used to identify frailty, although their relatively low specificity leads to false positive diagnoses and can therefore not be recommended as single measurements for detecting frailty [24]. Furthermore, because both gait speed and TUG consist of a single time-based value, a more detailed interpretation on reasons for the development of functional decline and, eventually, frailty, remains difficult. Botolfsen et al. (2008) evaluated a video-based analysis of all sub-tasks of the TUG [32]. They found higher levels of measurement precision by analysing each sub-task separately. However, their approach made the analysis of the TUG very time-consuming and thus not practicable in clinical settings. With the built-in sensor array used in the aTUG chair, we were able to provide a real-time data analysis of time-based results (time to complete the TUG and gait speed) as well as additional gait parameters such as cadence and step length. Our results show that the aTUG is able to provide additional information on mobility and gait parameters in a single test that is quick and easy to perform and is widely accepted in clinical settings for the identification of frailty. This potentially leads to new opportunities to include such parameters into frailty screening procedures to detect early signs of frailty for clinicians and therapists alike. The exact content of such parameters that are relevant for the detection of frailty have to be established in further research.

While gait speed is an established parameter that is associated with factors such as disability, cognitive decline, falls, and mortality [22], new evaluation concepts of gait such as the Walk Ration (WR) [39] include cadence in their calculation. Based on this research, the WR can detect older people with a risk of falling who show normal gait speed. Step length, on the other hand, is associated with functional loss and falls [40]. This demonstrates the potential benefits of including additional gait parameters in a mobility analysis of older people. However, while it seems obvious that current evidence on these gait parameters and their influence on functional ability, overall mobility and falls in older people can be exerted to the detection of frailty, no clear evidence for this exists to our knowledge. Schwenk et al. (2014) summarized in a systematic review the existing evidence and found promising, though not conclusive evidence on the potential of the use of new technologies for analysing gait in frail older people [62]. Therefore, while we conclude that our aTUG-system provides good evidence for the technical validity and feasibility of our system, future research is needed to evaluate which, if any, gait parameters can help researchers, clinicians, and therapists alike to identify frailty and to use them to document and control current functional status as well as changes over time due to further functional decline or rehabilitation. With the strong measurement validity of the aTUG and its timely measurement proceeding, the aTUG may be a more appropriate measurement system for addressing future research questions. Due to the overall measurement setup of the aTUG, we additionally see some advantages of our system in comparison to the actual gold standard, the GAITRite^®^ system. While the GAITRite^®^ has a very high measurement precision and has been proven to be able to measure several important gait parameters, its use is more time consuming. Additionally, the aTUG chair is highly mobile and can be quickly setup in different locations, e.g. in different rooms in a ward or home care setting. Furthermore, and most important, the aTUG chair provides measurements beyond gait speed and gait parameters, but also the TUG and, potentially, information on the different phases of the TUG as well as sitting balance. The validity of these additional information have to be confirmed in further studies. The same is true for establishing other validity measures such as test-retest reliability or minimal detectable change.

However, while many of the analysed gait parameters revealed good to excellent concordance with the reference standard, some of them were much lower. This merits some discussion. CC-values for stance time were good, though not excellent in our analysis. As the LRS of the aTUG was positioned under the seating of the chair, stance had to be measured indirectly as the LRS was not able to measure foot/floor-contact directly. Instead, the end of the stance time (and thus, start of the swing time) for each leg was derived from the onset of calf movement in the measured axis. Therefore, stance time could be either under or overestimated, depending on walking style. Still, measurement concordance with the reference standard was satisfying. For swing time, the described limitations of the LRS-measurements are comparable, but its consequences for measurement precision were obviously more severe. We therefore must conclude that until further development of the aTUG and/or its underlying algorithms, these measurements are not meeting the necessary requirements to be included in a gait analysis. In contrast, while correlations of total distance walked between aTUG and GAITRite^®^ were even lower, this most likely was the consequence of the different measurement technologies used in our study. In contrast to TUG-measurements, participants did not turn around and walked back to a defined point but rather extended the walking phase to the end of the GAITRite^®^. This adjustment was necessary as the GAITRite^®^ was only able to measure unidirectional walks and the turning and walk-back phases could not be recorded by the GAITRite^®^. Therefore, distance measurements exceeded the 3-meter range for which the LRS was calibrated. Because of the changes in measurement setup, another problem could potentially be responsible for the low CC we observed for the measured distance. As previously described, the GAITRite^®^ consists of physical capacitive sensors. Because of this, every measurement has a definite end when a participant steps down from the mat. In contrast, the LRS measurements are influenced by its range as well as its ability to detect reflections from the test subjects’ legs. In our measurements, we observed that the LRS had a much longer range than the GAITRite^®^, though this could potentially be influenced by additional factors such as lighting. We therefore took every effort to standardize the measurements as much as possible (including lighting). For this reason, we think that the lack of concordance of the distance measurements are due to the different measurement approaches of the two systems. Therefore, we consider this mainly a problem of the measurement setup that does not reflect higher measurement errors while performing a TUG. However, this should be evaluated with a suitable measurement setup in future studies. Finally, while most measurements went according to protocol, we had to exclude 12.7% of our measurements. While this number is rather high, the majority of these cases were due to either failure of the synchronisation tool, operation failure by the assessors or a lack of interpretable data from the GAITRite^®^. Still, the dependability of the aTUG chair still has to be improved in order to be a technically reliable instrument. The data and experience we gained in the study presented here have already been used in further development steps and, hopefully, we will be able to demonstrate improvements of the applicability of the aTUG chair in future publications.

In addition to the technical validation of the aTUG regarding its ability to measure gait parameters, we compared results from the GAITRite^®^ and the aTUG for several parameters (cadence, velocity, test duration, and step length) with results from the BBS and ABC assessments. To the best of our knowledge, this is the first publication comparing specific gait parameters with results from Balance and Balance-confidence measurements. In a recent review by Osaba et al. (2019), they reported several mechanisms that negatively influence both balance and gait [63]. This relationship is reflected in our results, where we found an association of better walking ability (represented by the measured gait parameters) with better balance (BBC) and Balance Confidence (ABC) scores via linear regression. While both scales demonstrated only a medium-sized correlation with velocity, cadence, and step length, this is hardly surprising. The BBS incorporates both static and dynamic balance measures and thus, only partly reflects functional requirements for walking. For balance confidence, this effect was even more pronounced. While FoF is more prevalent in frail older persons [13], the development of FoF is associated with multidimensional factors such as gender, age, and subjective health [64] as well as prior falls experience [65]. Therefore, gait parameters can only partly reflect these underlying mechanisms. In this, the clear and significant linear correlation between the measured gait parameters demonstrates the importance of measuring FoF in older people and especially in those at risk of falling. We see, however, potential opportunities to combine results from questionnaires measuring FoF or balance confidence with the aTUG to be able to evaluate functional reasons or consequences deriving from FoF. Again, additional research is necessary to establish any existing linkage.

The number and descriptive statistics of participants in the study show a balanced section of the target group for the aTUG system. We deliberately chose to include participants with existing functional limitations, but with a wide range of age. As a result, we deem the results presented here to be representative for the purpose of measuring the gait properties of elderly persons. Based on these results, we conclude that the measurement of different gait variables using a LRS is a viable and valid method to measure gait parameters such as gait speed, cadence, and step length, which are important parameters to assess mobility and walking ability in older people.

## Figures and Tables

**Table 1:** Considered gait parameters and corresponding algorithms; based on [50,52].

Gait Parameter	Description	Algorithm	Separately Per Foot
Step length [m]	Distance between the toe of one foot to the heel of the next one along walking direction (see left and right step in Figure 2)	distance passed from toe off to initial contact.	x
Cadence [1/min]	Step frequency	number of steps/time [min]	
Velocity [m/min]	Gait speed	distance [m]/time [min] = stride_length [m] × cadence [1/min]/ 2	
Stride length [m]	Distance from one foot hitting the ground to its next ground contact (see full gait cycle shown in Figure 2 as example for right stride).	step_lengthLeft + step_lengthRight	x

**Table 2:** Descriptive statistics of the 91 participants (64 females) with valid recordings.

	Mean	STD	Min	Max
Age (years)	73.5	6.8	58	92
Height (cm)	166.2	8.6	150	185
Weight (kg)	72.9	14.7	45	111
BMI	26.4	4.8	16.9	41.6
BBS	45.2	8.1	22	56
ABC	34.7	8.3	6	48

**Table 3:** Error metrics of the gait parameters calculated by the aTUG chair compared to GAITRite^®^ using the Pearson correlation coefficient.

	Error							CC
Parameter	Mean	Std	Min	25%	50%	75%	Max	
Velocity (m/s)	0.034	0.036	0.001	0.01	0.022	0.046	0.227	0.992
Stride Length Left (m)	0.022	0.020	0	0.007	0.018	0.033	0.108	0.992
Stride Length Right (m)	0.025	0.026	0.0003	0.008	0.018	0.032	0.145	0.989
Step Length Left (m)	0.020	0.018	0.000	0.006	0.015	0.029	0.096	0.976
Step Length Right (m)	0.019	0.017	0	0.007	0.015	0.027	0.091	0.977
Cadence (steps/min)	27.248	11.065	6.49	19.94	26.365	33.398	71.84	0.959
Steps (count)	3.094	1.985	0	2	3	4	9	0.820
Stance Time Left (m)	0.293	0.145	0.146	0.229	0.263	0.319	1.657	0.876
Stance Time Right (m)	0.290	0.193	0.047	0.225	0.273	0.317	2.324	0.798
Swing Time Left (s)	0.078	0.130	0.001	0.019	0.048	0.090	1.316	0.607
Swing Time Right (s)	0.071	0.079	0	0.025	0.050	0.098	0.720	0.708

**Table 4:** aTUG and GAITRite^®^ Correlation with BBS and ABC Score.

Score	Parameter	Sensor	Slope	Intercept	R Value	*p*-Value	Std-Error
BBS	cadence	aTUG	1.995	37.837	0.620	0.000	0.205
		GAITRite	1.261	43.963	0.595	0.000	0.132
	velocity	aTUG	0.029	−0.367	0.782	0.000	0.002
		GAITRite	0.028	−0.362	0.798	0.000	0.002
	step length R	aTUG	0.010	0.087	0.742	0.000	0.001
		GAITRite	0.010	0.097	0.729	0.000	0.001
	step length L	aTUG	0.011	0.024	0.704	0.000	0.001
		GAITRite	0.011	−0.002	0.743	0.000	0.001
	stride length R	aTUG	0.021	0.108	0.738	0.000	0.002
		GAITRite	0.021	0.093	0.756	0.000	0.002
	stride length L	aTUG	0.021	0.103	0.750	0.000	0.002
		GAITRite	0.021	0.096	0.757	0.000	0.002
ABC	cadence	aTUG	1.288	84.270	0.395	0.000	0.238
		GAITRite	0.837	72.527	0.408	0.000	0.152
	velocity	aTUG	0.020	0.241	0.530	0.000	0.003
		GAITRite	0.020	0.231	0.541	0.000	0.002
	step length R	aTUG	0.007	0.294	0.484	0.000	0.001
		GAITRite	0.007	0.294	0.478	0.000	0.001
	step length L	aTUG	0.007	0.268	0.459	0.000	0.001
		GAITRite	0.007	0.265	0.487	0.000	0.001
	stride length R	aTUG	0.015	0.561	0.491	0.000	0.002
		GAITRite	0.014	0.560	0.497	0.000	0.002
	stride length L	aTUG	0.015	0.563	0.487	0.000	0.002
		GAITRite	0.014	0.568	0.487	0.000	0.002

## Data Availability

Not Applicable.

## Abbreviations

Abbreviations used in this manuscript:

ADL	activities of the daily living
aTUG	ambient TUG
CC	correlation coefficient
FS	force sensor
IQR	interquartile range
LB	light barrier
LRS	laser range-scanner
SD	standard deviation
TUG	Timed “Up & Go”
